# Endoscopic biliary drainage using a narrow-diameter endoscope in a patient with obstructive jaundice and pancreatic cancer

**DOI:** 10.1055/a-2418-1018

**Published:** 2024-10-08

**Authors:** Koichi Soga, Mayumi Yamaguchi, Takeshi Fujiwara, Fuki Hayakawa, Yumi Kusano, Ikuhiro Kobori, Masaya Tamano

**Affiliations:** 1 26263 Department of Gastroenterology, Dokkyo Medical University Saitama Medical Center, Koshigaya, Japan

Standard endoscopic retrograde cholangiopancreatography (ERCP) involves the insertion of a duodenoscope and side-viewing endoscope. However, advancement of the scope into the correct gastrointestinal lumen can be challenging in patients with a deformed superior duodenal angle caused by peritoneal dissemination or direct tumor invasion 1 .

A 68-year-old Japanese woman presented with cholangitis and obstructive jaundice secondary to pancreatic cancer. Two months before presentation, a biliary plastic stent was placed to alleviate obstructive jaundice. Further examination revealed that the stent had migrated deep into the duodenum, thereby necessitating replacement. Initially we attempted to insert a side-viewing endoscope into the duodenum; however, it would not pass through the duodenal stenosis. Therefore, we attempted endoscopic ultrasound-guided hepaticogastrostomy. Although biliary needle puncture was successful, the guidewire could not be advanced to the deeper bile duct ( Fig. 1 , Fig. 2 ).

**Fig. 1:**
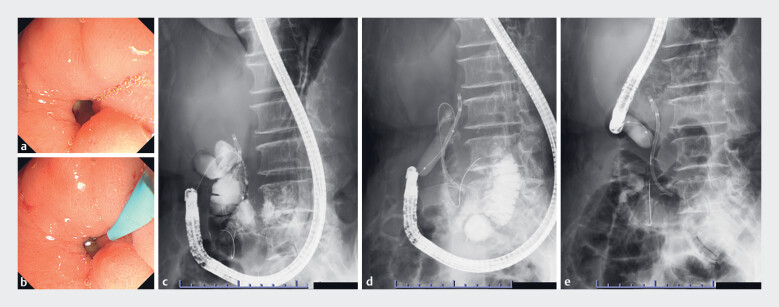
Endoscopic retrograde cholangiopancreatography (ERCP) with existing endoscopic equipment. **a** We attempted to insert a side-viewing endoscope in the duodenum; however, the endoscope could not be passed through the stenosis at the first part of the duodenum. **b, c** We attempted to insert a side-viewing endoscope deep in the duodenum using an ERCP cannula and guidewire. **d,e** We attempted to insert a side-viewing endoscope deep into the duodenum after using a balloon to perform gastrointestinal dilation; however, deep insertion could not be achieved.

**Fig. 2:**
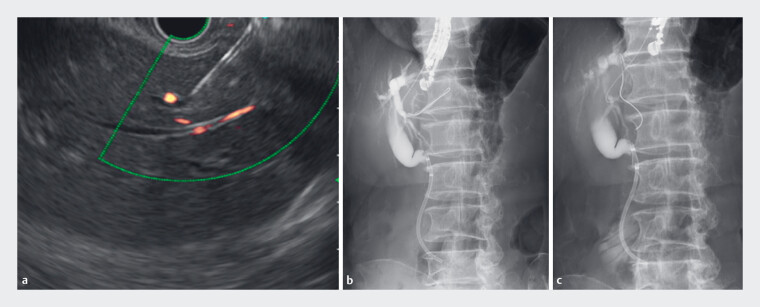
Endoscopic ultrasound-guided hepaticogastrostomy procedures with existing endoscopic equipment. **a, b** We attempted endoscopic ultrasound-guided hepaticogastrostomy of the stomach. Biliary puncture with a 19-gauge needle was successful. **c** The guidewire could not reach the central bile duct; therefore, this drainage approach was abandoned.

A week later, we attempted endoscopic drainage using a novel thin endoscope (EG-840TP; Fujifilm, Tokyo, Japan) with an outer diameter of 7.9 mm and forceps channel diameter of 3.2 mm. Despite its smaller diameter, the larger forceps channel offers various advantages for endoscopy 2 . We successfully navigated through the duodenal stenosis and reached the descending duodenum with the thin endoscope. Then, we positioned the endoscope perpendicular to the duodenal papilla using an endoscopic hood. By using the existing plastic stent as a marker, we aligned the guidewire coaxially with the stent using an endoscopic sphincterotomy (ES) knife (ENGETSU; Kaneka, Tokyo, Japan). This allowed the guidewire to enter the common bile duct and enabled insertion of an ERCP cannula. After cholangiography, a plastic stent (7 Fr × 12 cm; SUZAKU; Kaneka) was successfully placed ( Fig. 3 , Fig. 4 ; Video 1 ).

**Fig. 3:**
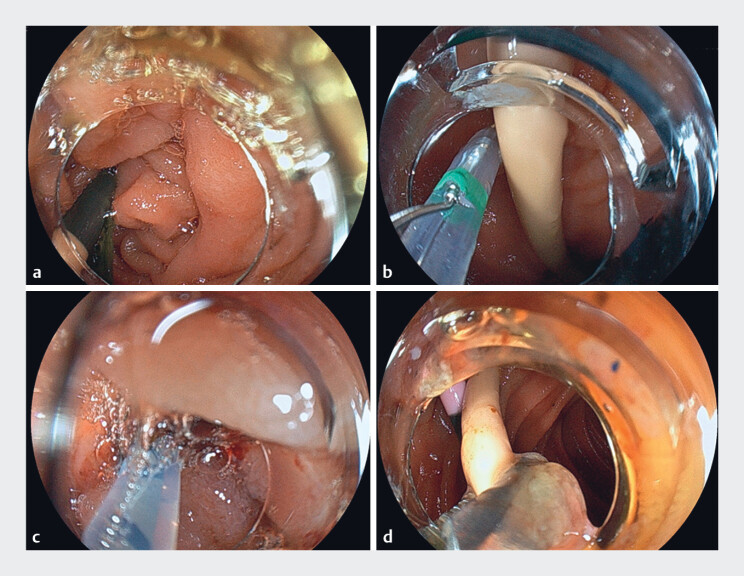
Endoscopic images of endoscopic biliary drainage using a narrow-diameter endoscope and endoscopic sphincterotomy (ES) knife. **a** We attempted endoscopic drainage using a novel thin endoscope with an outer diameter of 7.9 mm and forceps channel diameter of 3.2 mm. Using the thin endoscope, we successfully navigated through the duodenal stenosis and reached the descending duodenum. **b** With the existing plastic stent as a marker, we aligned the guidewire coaxially with the stent using a novel ES knife. **c** An endoscopic retrograde cholangiopancreatography cannula was inserted into the bile duct. **d** After cholangiography, a plastic stent was successfully placed.

**Fig. 4:**
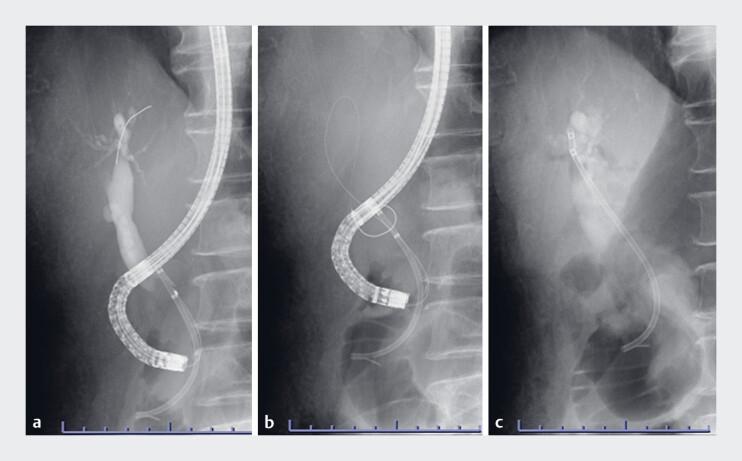
Fluoroscopic images of endoscopic biliary drainage using a narrow-diameter endoscope and endoscopic sphincterotomy (ES) knife. **a** We positioned the endoscope perpendicular to the duodenal papilla using an endoscopic hood on the endoscope tip. **b** This approach allowed the guidewire to follow the path of the plastic stent, and an endoscopic retrograde cholangiopancreatography cannula was inserted in the bile duct. **c** We removed the existing stent using grasping forceps. A plastic stent was successfully placed.

Endoscopic biliary drainage was performed using a narrow-diameter endoscope and endoscopic sphincterotomy knife.Video 1

This case demonstrates the effectiveness of advanced endoscopic procedures and stent placement using a novel thin endoscope and ES knife for duodenal stenosis.

Endoscopy_UCTN_Code_TTT_1AR_2AK

## Notes

Endoscopy E-Videos https://eref.thieme.de/e-videos
*E-Videos* is an open access online section of the journal *Endoscopy* , reporting on interesting cases and new techniques in gastroenterological endoscopy. All papers include a high-quality video and are published with a Creative Commons CC-BY license. Endoscopy E-Videos qualify for HINARI discounts and waivers and eligibility is automatically checked during the submission process. We grant 100% waivers to articles whose corresponding authors are based in Group A countries and 50% waivers to those who are based in Group B countries as classified by Research4Life (see: https://www.research4life.org/access/eligibility/ ). This section has its own submission website at https://mc.manuscriptcentral.com/e-videos .
